# Mitochondrial Dynamics in Adult Cardiomyocytes and Heart Diseases

**DOI:** 10.3389/fcell.2020.584800

**Published:** 2020-12-17

**Authors:** Anqi Li, Meng Gao, Wenting Jiang, Yuan Qin, Guohua Gong

**Affiliations:** ^1^Institute for Regenerative Medicine, Shanghai East Hospital, School of Life Sciences and Technology, Tongji University, Shanghai, China; ^2^Department of Pharmacy, Shanghai East Hospital, Tongji University, Shanghai, China

**Keywords:** heart, mitochondrial fusion, mitochondrial fission, dynamics, mature cardiomyocytes

## Abstract

Mitochondria are the powerhouse organelles of cells; they participate in ATP generation, calcium homeostasis, oxidative stress response, and apoptosis. Thus, maintenance of mitochondrial function is critical for cellular functions. As highly dynamic organelles, the function of mitochondria is dynamically regulated by their fusion and fission in many cell types, which regulate mitochondrial morphology, number, distribution, metabolism, and biogenesis in cells. Mature rod-shaped cardiomyocytes contain thousands of end-to-end contacted spheroid mitochondria. The movement of mitochondria in these cells is limited, which hinders the impetus for research into mitochondrial dynamics in adult cardiomyocytes. In this review, we discuss the most recent progress in mitochondrial dynamics in mature (adult) cardiomyocytes and the relationship thereof with heart diseases.

## Introduction

Mitochondria are essential subcellular organelles in most eukaryotic cells. They are composed of an outer membrane, small intermembrane space, an inner membrane containing an electron transport chain (ETC), and a matrix retaining multiple copies of their genome (Vignais et al., 1969; Koch et al., 2017). Mitochondria provide energy in the form of adenosine triphosphate (ATP) for cell activities. ATP is produced through the tricarboxylic acid (TCA) cycle and the oxidative phosphorylation system (OXPHOS) in mitochondria (Rambold and Pearce, 2018). Logically, power-hungry cells require more mitochondria to keep up with energy demand than do cells with lower energy needs. In adult cardiac cells, more chemical energy is consumed by excitation and contraction than in other non-contractile cells. An adult human heart will consume ca. 6 kg ATP per day to pump blood (Mishra et al., 2017; Steggall et al., 2017). Consequently, mitochondria, which produce 90% of the cell's ATP, occupy ca. 40% of the cell volume of adult cardiac cells (Doenst et al., 2013).

Although energy production plays a central role in mitochondria, they are also involved in multiple cellular functions, such as free radical production, calcium homeostasis, cell apoptosis, and necrosis (Nunnari and Suomalainen, 2012; Koch et al., 2017). Mitochondria are very sensitive to stress. Dysfunctional mitochondria have frequently been observed in various diseases, including neurodegenerative diseases, diabetes, and heart diseases (Krieger and Duchen, 2002; Nunnari and Suomalainen, 2012; Rambold and Pearce, 2018). Therefore, mitochondrial function must be well-controlled to avoid cell dysfunction or death. Multiple mechanisms, including mitochondrial biogenesis and mitochondrial autophagy, have been utilized by cells to maintain mitochondrial homeostasis (Westermann, 2010; Zhang and Xu, 2016; Seung-Min and Yong-Keun, 2018).

**Figure 1:**
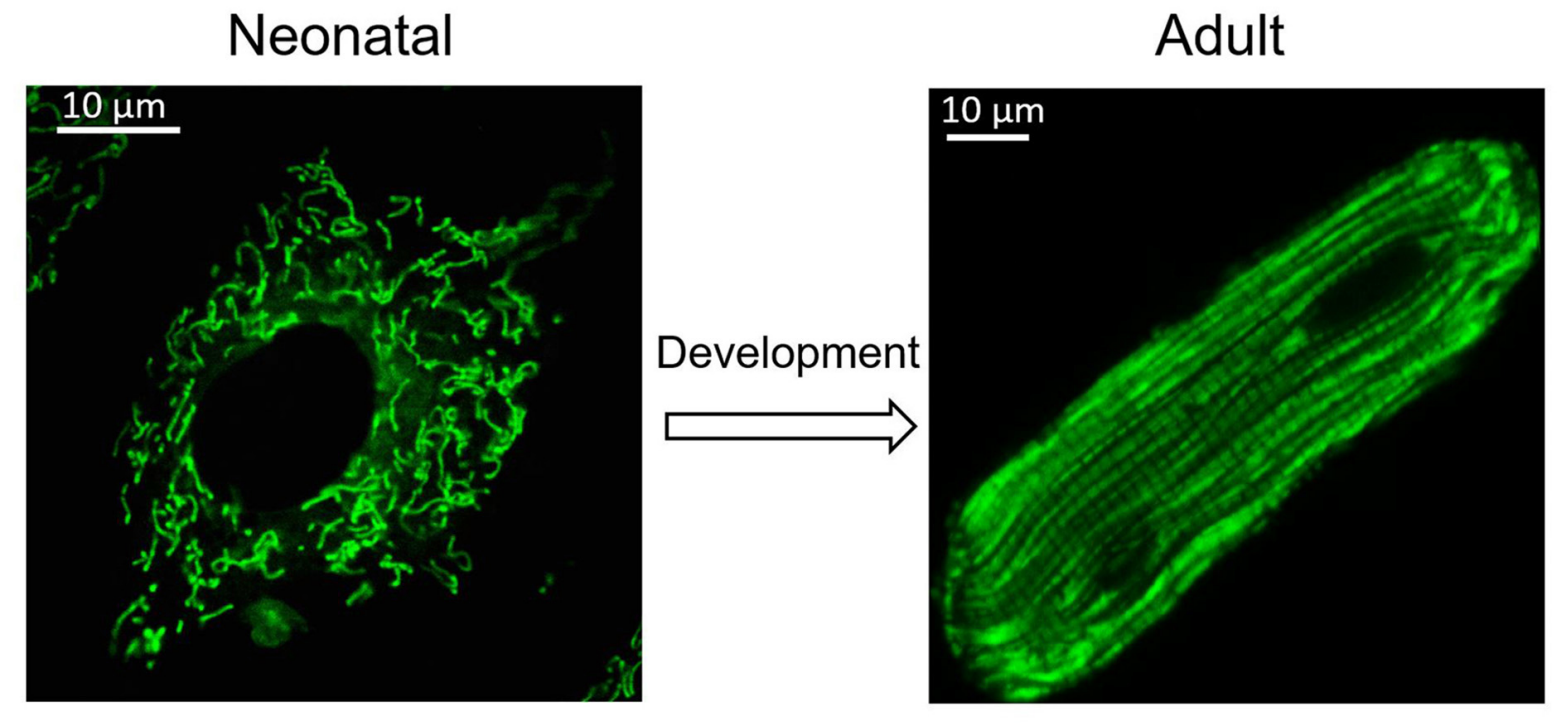
Change of mitochondria during heart development. Cardiomyocytes undergo morphological changes during heart development. Mitochondria also adjust their location and morphology during heart development. Spheroid mitochondria replace the fused mitochondrial network seen in immature cardiomyocytes after cardiomyocyte maturation.

Many other mitochondrial characteristics, such as their location and morphology within the cell, are also essential to maintain mitochondrial function. Mitochondrial dynamics was proposed by Lewis and Lewis (1914). They found that mitochondria can change shape when the cell is stimulated (Lewis and Lewis, 1914). Changes in mitochondrial shape are related to crucial cellular functions, including reactive oxygen species (ROS) and Ca^2+^ signaling (Yu et al., 2006; Hom et al., 2010). Any modification of the morphology and the internal matrix composite of mitochondria could impair mitochondrial functions and contribute to cell dysfunction or death (Bereiter-Hahn and Vöth, 1994). Mitochondria are constantly moving and undergoing shape changes controlled by mitochondrial fusion and fission in many cells (Detmer and Chan, 2007). In fibroblasts and many other types of cells, mitochondria can easily adjust their location and morphology, depending on metabolic conditions and energy needs. Generally, active metabolic cells with a fused mitochondrial network are quiescent cells with fragmented mitochondria (Rafelski, 2013). However, in the adult heart, active metabolic cardiomyocytes exhibit a fragmented mitochondrial network (Figure 1) (Wai et al., 2015). In adult cardiac cells, there are many myofilaments and a rigid cytoskeleton. Spheroid mitochondria are densely confined among myofibrils to ensure quick and efficient energy fluxes (Tepp et al., 2011). This type of arrangement limits the movement of mitochondria. Although mitochondrial dynamism proteins that mediate mitochondrial fusion and fission are abundantly expressed in adult cardiac, fragmented mitochondria appear to be frozen in adult cardiomyocytes (Vendelin et al., 2005). Given that proteins related to mitochondrial dynamics are involved in multiple processes of cardiac physiology, in addition to their role in mitochondrial fission and fusion, it is thought that adult cardiomyocyte mitochondria are hypodynamic. However, recent evidence indicates that adult cardiomyocytes may be less static than first believed (Song and Dorn, 2015; Eisner et al., 2017).

In this review, we focus on the current understanding of mitochondrial dynamics and their role in cell function and heart diseases, with a fresh perspective in adult cardiomyocytes.

## Mitochondrial Dynamics Proteins in Adult Cardiac Myocytes

In order to maintain cellular homeostasis, mitochondrial quality must be controlled appropriately. Damaged mitochondria are divided into two daughter mitochondria, one healthy and the other unhealthy, by fission. The healthy mitochondrion can fuse with other healthy mitochondria to exchange lipid membranes and intramitochondrial content. The sick mitochondrion is removed by lysosome-mediated mitophagy (Dorn, 2013). Normal mitochondria can also undergo fission to generate two healthy daughter mitochondria for metabolic regulation. Mitochondrial fusion and mitochondrial fission involve major mitochondrial dynamics, regulating the shape, length, and number of mitochondria. Peroxisome proliferator-activated receptor gamma co-activator 1-alpha (PGC-1α) is responsible for mitochondrial biogenesis. This protein can also influence mitochondrial fission/fusion by regulating mitochondrial fusion and fission protein expression (Peng et al., 2017). Inhibiting mitochondrial fission or promoting mitochondrial fusion can promote mitochondrial biogenesis (Peng et al., 2017).

**Figure 2:**
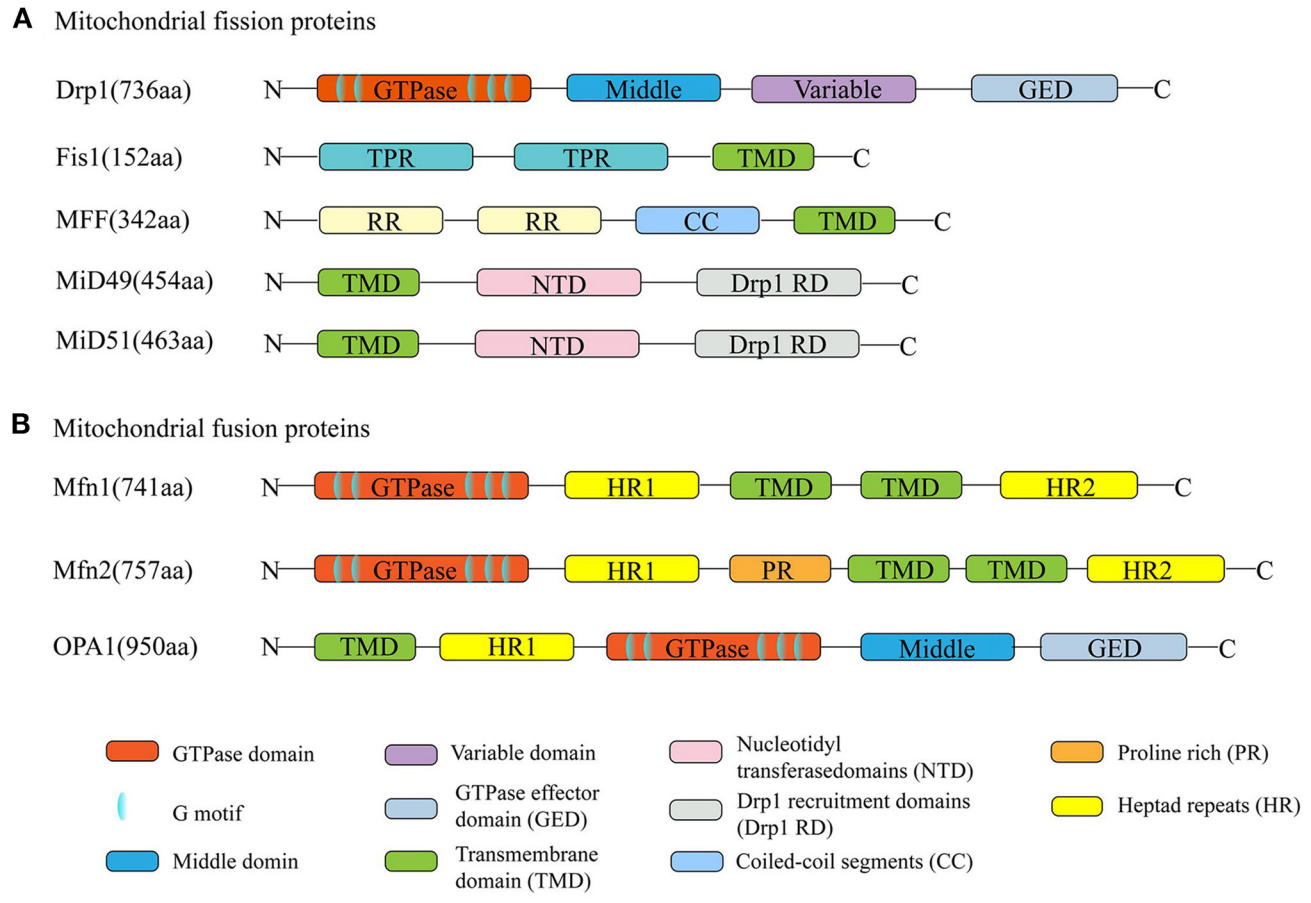
Schematic diagrams of mitochondrial dynamism proteins. **(A)** The dynamin-related protein 1 (Drp1) is the major mitochondrial fission protein, with GTPase activity. The anchored adapter proteins, fission protein 1 (Fis1), mitochondrial fission factor (MFF), and mitochondrial dynamics proteins of 49 kDa and 51 kDa (MID49/51) also participate in the fission process. **(B)** Mitofusin 1/2 (Mfn1/2) and optic atrophy 1 (OPA1) mediate the mitochondrial fusion process. Their normal functions rely on the activity of GTPases. The structures of Mfn1 and Mfn2 are similar. They have about 80% sequence similarity.

Mitochondrial fission is mediated by dynamin-related protein 1 (Drp1); the outer mitochondrial membrane (OMM)-anchored adapter protein, fission protein 1 (Fis1); mitochondrial fission factor (MFF); and mitochondrial dynamics proteins of 49 kDa and 51 kDa (MID49/51) (Kraus and Ryan, 2017). Mitochondrial fusion is mediated by the OMM proteins mitofusin 1/2 (Mfn1/2) and the inner mitochondrial membrane (IMM) protein optic atrophy 1 (OPA1) (Song et al., 2009). All these proteins are nuclear-encoded and are abundantly expressed in the adult heart (Figure 2). Their normal functions rely on the activity of guanosine triphosphatases (GTPases) (Hoppins et al., 2007).

### Mitochondrial Fission Proteins

Of all the proteins involved in mitochondrial division, Drp1 is the key operator mediating mitochondrial fission. Its structure and function are well-understood. There are six Drp1 isoforms in humans, which are generated by alternative splicing. These isoforms show tissue specificity. Drp1 isoform 4 is weakly expressed in the brain, heart, and kidney, while isoform 5 (710 amino acids) occurs mainly in the heart, liver, and kidney. As the master fission operator, Drp1 has a higher-order structure. Like other dynamin family members, it has an N-terminal GTPase domain, followed by the middle domain, variable domain, and the GTPase effector (GED) domain in the C-terminus. These four domains interact with each other to form multimers and perform different functions (Muhlberg et al., 1997; Takei et al., 1998; Smirnova et al., 1999). For example, GTPase activity is activated by a combination of the GED and GTPase domains (Muhlberg et al., 1997). Posttranslational modifications of Drp1 amino acid residues are strongly associated with Drp1 recruitment, construction, and activity (Chang and Blackstone, 2007a, 2010; Taguchi et al., 2007; Santel and Frank, 2008). For example, phosphorylation of Ser^637^ or Ser^656^ by cAMP-dependent protein kinase A (PKA) inhibits Drp1 GTPase activity and affects mitochondrial fission (Chang and Blackstone, 2007a; Taguchi et al., 2007). Conversely, dephosphorylation of Ser^637^ promotes Drp1 translocation to the mitochondria (Chang and Blackstone, 2007b). However, it is puzzling that different protein kinase modifications at the same site have different results, and the same protein kinase modifications at different sites produce opposite effects. It has been demonstrated that when Ca^2+^/calmodulin-dependent protein kinase Iα (CaMKIα) phosphorylates Drp1 at Ser^637^, Drp1 GTPase activity is activated, which is opposite to the effect of phosphorylation at this site by PKA (Han et al., 2008). Similarly, phosphorylation of Ser^616^ of Drp1 by extracellular signal-regulated kinase (ERK) activates it, while phosphorylation at this site by cyclin-dependent kinase (CDK) inhibits its activity (Cho et al., 2014; Kashatus et al., 2015).

Under physiological conditions, most Drp1 exists in the cytoplasm, in equilibrium between dimeric and tetrameric forms. Approximately 3% of Drp1 is distributed at the mitochondrial surface, as detected by subcellular fractionation and Western blot experiments (Smirnova et al., 2001). The lack of a membrane-spanning domain makes Drp1 unable to participate directly in mitochondrial fission; hence, Drp1 adaptors, which help Drp1 to move to the constriction site to exert fission, are indispensable to the process. The mitochondrial adapter Fis1 and MFF are two receptors of Drp1 (Yoon et al., 2003; Gandre-Babbe and Van Der Bliek, 2008). Fis1 was the first OMM receptor identified as recruiting Drp1 and is thought to bind to Drp1, forming a copolymer *via* its two tetratricopeptide repeat (TPR) domains (Suzuki et al., 2003). The function of this copolymer remains controversial. Elevated expression of Fis1 induces Drp1 movement from the cytosol to the OMM, while inhibition of Fis1 shows unaffected Drp1 translocation (Lee et al., 2004). Another Drp1 receptor, MFF, consisting of 342 amino acids, is located in the OMM, facing the cytosol. Unlike Fis1 inhibition, knockdown of MFF leads to decreased recruitment of Drp1 protein to mitochondria (Otera et al., 2010). Both MiD49 and MiD51 contain nucleotidyl transferase (NT) domains and DRP1 recruitment domains (Drp1 RDs) (Kalia et al., 2018). However, it remains unclear whether it promotes Drp1 movement during fission due to the paradoxical results in overexpression and knockdown of either or both MiD proteins (Losón et al., 2013).

Mitochondrial fission is a multistep and complex process, along with GTP hydrolysis (Van Der Bliek et al., 2013). The division process involves constriction of both the OMM and IMM. IMM constriction is poorly understood, while OMM division has been well-studied. Matrix and mitochondrial DNA (mtDNA) are redistributed during mitochondrial division. At the beginning of the fission activity, mtDNA is replicated in the mitochondrial matrix. The replication occurs at mitochondrion–endoplasmic reticulum (ER) contact sites, which participate in initiating OMM constriction and drive the fission process (Friedman et al., 2011). Recently, structural biological studies have shown that the Drp1 neck (ca. 100 nm) is much thinner than the mitochondrial diameter (0.5–1.0 μm); therefore, Drp1 is unable to initiate mitochondrial constriction, implying that there is another step before Drp1 (Mears et al., 2011). IMM constriction occurs at mitochondrion–ER contacts in a Ca^2+^-dependent process before Drp1 oligomerization and maturation (Cho et al., 2017).

Drp1 exists as tetramers and dimers in the mitochondrial surface and cytosol. The formin-family protein, inverted formin 2 (INF2), which is anchored in the ER, cooperates with the mitochondrial Spire1C to mediate actin polymerization and nucleation at mitochondrion–ER contact sites, which is a critical step before Drp1 recruits mitochondria (Korobova et al., 2013). Non-muscle myosin II is found around mitochondria and may build and constrict a circumferential actin ring (Yang and Svitkina, 2019). Next, Drp1 proteins are recruited by MFF and MiDs to the mitochondrion–ER sites to form ringlike oligomers around the mitochondria; these are the sites at which constriction occurs, resulting in the generation of daughter mitochondria by the process of fission (Gandre-Babbe and Van Der Bliek, 2008; Palmer et al., 2011, 2013). Interestingly, there are some differences in the mechanism of Drp1 recruitment by MFF and MiDs. It is assumed that MFF selectively identifies oligomeric and active Drp1, while MiDs contact the GTP-bound state of Drp1 to promote oligomerization (Palmer et al., 2011; Liu and Chan, 2015). During this process, the conformation of Drp1 is changed by GTP hydrolysis (Hoppins et al., 2007; Mears et al., 2011). After Drp1 induces membrane constriction, the canonical Dnm2 protein assembly at the Drp1 neck is involved in the final membrane scission (Hoppins et al., 2007; Kraus and Ryan, 2017). Upon GTP hydrolysis, further constriction occurs to generate the two new daughter mitochondria.

### Mitochondrial Fusion Proteins

Generally, mitochondrial fission is rapidly followed by a fusion event. Mitochondrial fusion involves OMM and IMM fusion, induced by Mfn1/2 and OPA1, respectively. The structures of Mfn1 and Mfn2 are similar, and the proteins have about 80% sequence similarity (Chen et al., 2003). Both proteins are anchored in the OMM through their two transmembrane (TM) domains, which are separated by a short loop, exposing their N-terminal region, containing the GTPase domain with five specific functional motifs, the coiled-coil heptad repeat 1 (HR1) domain, and their C-terminal harboring a second coiled-coil HR2 domain located in the intermembrane space (IMS). Compared with Mfn1, Mfn2 expression is more abundant in heart and muscle tissues than in other tissues (Santel et al., 2003). Additionally, Mfn2 harbors a proline-rich domain that does not exist in Mfn1 and may be involved in protein–protein interactions, suggesting that the protein has an important function (Ranieri et al., 2013). In cardiac myocytes, Mfn2, as a mitochondrion–ER tether protein, mediates transfer of calcium and other small molecules between the mitochondria and ER (Dorn, 2020).

OPA1 is a crucial element in IMM fusion and is located in the IMM *via* an N-terminal matrix targeting signal, followed by a TM domain. The GTPase and GTPase effector domains in OPA function in GTP hydrolysis during fusion. It has been reported that mitochondrial fusion is abolished by inhibition of GTPase activity, indicating that GTPase activity, such as GTP hydrolysis, is crucial to this process (Hales and Fuller, 1997; Hermann et al., 1998). Moreover, OPA1 has five isoforms, including two higher molecular weight forms (long-form), referred to as L-OPA1, and three short-form soluble forms, known as S-OPA1 (Ishihara et al., 2006).

The fusion event is closely associated with the topology of Mfns (Mattie et al., 2017). First, the OMMs of two adjacent mitochondria tether each other *via* the HR1 and HR2 domains of Mfns (Chandhok et al., 2018). GTP hydrolysis induces a conformational change in Mfns; then, the two membranes dock with each other, resulting in increased contact surface area and the decreased distance between the two membranes (Escobar-Henriques and Anton, 2013). Finally, the fusion of the two OMMs is completed through a GTPase-dependent power stroke. Whether IMM fusion follows the OMM fusion or occurs concurrently with the OMM fusion has been studied for many years. Based on drug assays, Malka et al. (2005) proposed that the IMM fusion event is independent of OMM fusion. The IMM fusion process is regulated by OPA1 and specific IMM lipid components, particularly cardiolipin (CL). CL is a phospholipid that is important for maintaining the stability of large protein complexes, such as OXPHOS complexes and the ETC complexes that are involved in energy production (Ban et al., 2017). L-OPA1 interacts directly with CL to drive IMM fusion (Ban et al., 2017). S-OPA1 was found to enhance the interaction between L-OPA1 and CL (Devay et al., 2009). The proteolytic function of OPA1 is crucial for the induction of IMM fusion (Ban et al., 2017). After fusion, Mfn2 and OPA1, as membrane-bound proteins, are disassembled.

## Mitochondrial Dynamics in Adult Cardiomyocytes

### Change in Mitochondria During Heart Development

Mitochondria, as energy factories, are essential organelles for myocardial development. Intriguingly, mitochondria are also needed for developmental transitions related to the changes in the availability of nutrients and oxygen to the heart after birth (Gong et al., 2015a; Gottlieb and Bernstein, 2015). During heart development, bioenergetics change from anaerobic glycolysis to oxidation of fatty acids (the main fuel of the heart) (Ingwall and Weiss, 2004). In early embryonic development, the heart begins to form and demands little oxygen; thus, anaerobic glycolysis is the primary way to obtain energy (Porter et al., 2011). As the placenta matures and blood circulation is established, the level of oxidation increases, and cell metabolism changes to aerobic respiration for generation of ATP (Burton, 2009). Moreover, changes in the source of productivity substrates and alterations in metabolic states occur. Individual mitochondrial morphology and mitochondrial networks continuously change during the development of the heart and differentiation of cardiomyocytes, which is closely related to mitochondrial function (Gong et al., 2015a).

The sarcoplasmic structure of cardiomyocytes, including their mitochondria, is much simpler in the immature heart than in adult cardiomyocytes. At 10 days of gestation in the mouse, the IMM is relatively smooth and lacks mature pupae. At 9.5 days of gestational age, the mitochondrial network is dispersed, and mitochondria are mainly localized in the nucleus. However, at 13.5 days of gestational age, the mitochondrial network becomes dense and is cross-linked with each other, and mitochondria are also spread throughout the cell (Porter et al., 2011). Linear or long rod-shaped mitochondria are entirely replaced by spheroid mitochondria through Parkin-mediated mitophagy (a mitochondrial quality control pathway), and mitobiogenesis occurs in cardiomyocytes over the first 3 weeks after birth (Burton, 2009; Gong et al., 2015a; Gottlieb and Bernstein, 2015).

In mature cardiomyocytes, mitochondria have a unique limitation in distribution, rather than forming a network structure: (1) They are aligned in longitudinal rows between bundles of myofilaments (interfibrillar mitochondria, which are the most extensive form in skeletal muscle); (2) They are irregularly distributed under the sarcolemma (subsarcolemmal mitochondria); and (3) They are clustered on opposite sides of the nucleus (perinuclear mitochondria). Changes in mitochondrial structure and function reflect transformation of cardiomyocytes (Shimada et al., 1984; Barzda et al., 2005).

### Mitochondrial Dynamic Events in Adult Cardiomyocytes

Mitochondria are viewed as highly dynamic organelles that undergo continuous movement, fission, and fusion. This was first described in yeast 20 years ago and was found to be essential processes for maintaining healthy mitochondria (Hermann et al., 1998; Bleazard et al., 1999; Sesaki and Jensen, 1999). In HL-1 cardiac muscle cells, dense tubular mitochondria undergo continuous movement, fusion, and fission at a high velocity (90 nm/s) (Beraud et al., 2009b). However, mitochondria in adult cardiomyocytes lack motility, and the crystal arrangement of mitochondria in adult cardiomyocytes is very different from that in other types of cells and in rat fetal myocardial cells (Beraud et al., 2009a; Eisner et al., 2017). Movement is a prerequisite for mitochondria to exchange content frequently for regeneration and repair of impaired mitochondria through mitochondrial fusion and fission (Liu et al., 2009). For a long time, mitochondria were viewed as very important but static organelles, without fusion and fission, acting as energy factories in myocytes.

Green fluorescent protein (GFP), which can be excited by 488-nm light, has been used for photolabeling to visualize mitochondrial dynamics by means of confocal microscopy (Patterson and Lippincott-Schwartz, 2002). Beraud et al. (2009a) sought to identify mitochondrial interconnections using mitochondrial-targeted GFP in adult cardiomyocytes in 2009. Mitochondria showed very rapid fluctuations within the limited external space of mitochondria during 30 min of scanning, at 400-ms intervals, in non-beating HL-1 cells (Beraud et al., 2009a). However, evidence for mitochondrial fusion or fission in adult cardiomyocytes was absent.

**Figure 3:**
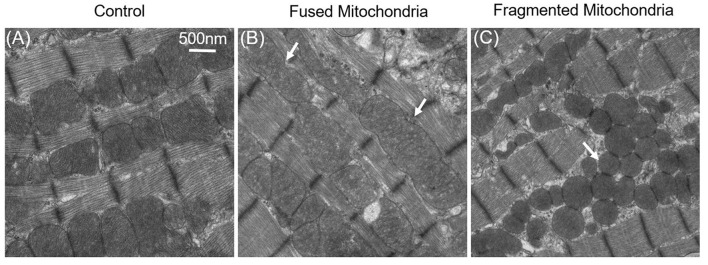
Fused and fragmented mitochondria in adult cardiomyocytes. **(A)** Normal mitochondria in the adult cardiac. **(B)** Fused mitochondria in adult cardiomyocytes with dynamin-related protein 1 (Drp1) inhibition; the white arrows indicate that the mitochondria were fused from 2–3 normal-sized mitochondria. **(C)** Fragmented mitochondria in the Mfn1/2 knockout adult cardiomyocytes: mitochondrial size is decreased, and mitochondria have become round.

It was therefore questioned whether mitochondrial dynamics exist in mature cardiac cells, even though the mitochondrial dynamics proteins are highly expressed in adult cardiac (Gong et al., 2015b). Although cardiac mitochondrial fusion and fission are not detected in cultured adult cardiomyocytes, it does not mean that these mitochondrial dynamics do not occur. Conversely, loss-of-function studies in the heart has shown that abnormal expression of mitochondrial dynamics proteins are closely connected with mitochondrial morphology and that this affects cardiac cellular functions (Figure 3) (Dorn Ii, 2015).

Study of mitochondrial dynamics using the traditional method of mitochondrial labeling in adult cardiomyocytes is important. A photoactivatable GFP (PAGFP), a form of GFP, can develop intense green fluorescence after activation by 405 nm laser light, from being nearly invisible, and its signal remains stable for days (Patterson and Lippincott-Schwartz, 2002, 2004; Lukyanov et al., 2005). It has been used to observe mitochondrial fusion and fission activities in adult cardiomyocytes through fluorescence decay within the photoactivation areas (Eisner et al., 2017; Zhang et al., 2017). Another photoactivatable mitochondrion-targeted fluorescent protein, MitoDendra, has also been used to monitor fusion (Lee et al., 2014). After laser stimulation of a small area of the mitochondrion, mitochondrial matrix-targeted PAGFP was activated by 488 nm light and emitted green fluorescence in this mitochondrion. After several hours, the activated PAGFP diffused to adjacent mitochondria, evidenced by the emergence of green fluorescence. Over time, mitochondria-activated PAGFP spread to more mitochondria and became more diluted (Huang et al., 2013; Eisner et al., 2017). This phenomenon represents putative fusion and fission events, with component exchange, between adjacent mitochondria. The distribution of activated GFP fluorescence intensity was inhomogeneous, suggesting that mitochondrial fusion was a selective process. Eisner et al. (2017) evaluated 24- to 48-h cultured neonatal ventricular cardiomyocytes (NVCMs). They found that PAGFP robustly spread in NVCMs and reached distant organelles within 5 min (Eisner et al., 2017) based on individual normalized fluorescence traces of 10 individual mitochondria every 20 s throughout the entire 20-min recording session. Quantitative analyses of the fusion events in freshly isolated adult ventricular myocytes (AVCMs) revealed a rate of 1.4 ± 0.1 events/min. All mitochondria in an adult cardiomyocyte form one dynamic but continuous network, exchanging both matrix and membranous components over a timescale of ca. 10 h.

It has been reported that some individual cardiac mitochondria are still interconnected in adult cardiomyocytes (Huang et al., 2013). Reports using mtPAGFP in adult cardiomyocytes have different findings. Instead of continuous mitochondrial fusion and fission, which require mitochondrial motility, structurally restricted mitochondria in adult cardiomyocytes exchange their contents (communication) by “kissing” and by nano-tunnels (Huang et al., 2013). Cheng lab proposed that pairs of adjacent, touching mitochondria show the same length and transfer their content disequilibrium; such an event is called kissing. In addition, two neighboring or remote mitochondria can communicate in a more conventional manner *via* a thin nano-tunnel structure connection, called nano-tunneling. In contrast to kissing, nano-tunneling is a saltatory event. *In vivo* experiments further confirmed this conjecture. Acute manipulation of fission and fusion proteins induced significant morphological changes in H9C2 cardiomyoblasts but only mild changes in adult cardiomyocytes.

Although putative fission and fusion, kissing, and nano-tunneling can mediate inter-mitochondrial communication (component exchange), real-time mitochondrial dynamic events in single mitochondria in mature cardiomyocytes have not yet been directly visualized.

**Figure 4:**
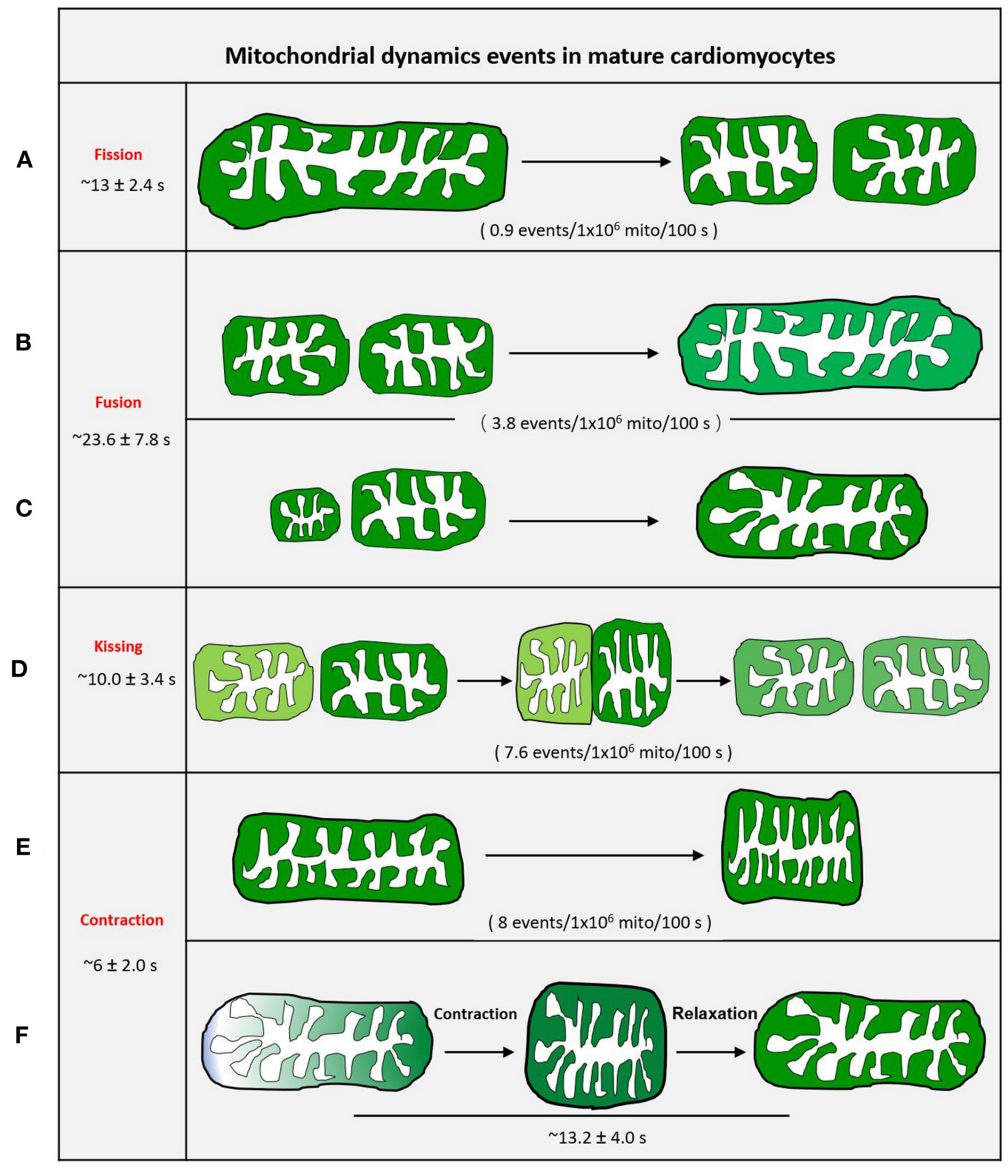
Schematic diagram of mitochondrial dynamic events of adult cardiomyocytes. **(A)** Mother mitochondria underwent contracting, stretching, and then splitting to generate two equal daughter mitochondria with slightly different fluorescence. **(B)** Two similar-sized adjacent mitochondria with different fluorescence touch and squeeze together for fusion, then stretch to normal. **(C)** The smaller mitochondrion moves toward the bigger mitochondrion and devotes itself to the big one. **(D)** Two adjacent individual mitochondria give a deep touch and quick separation to exchange the content as indicated by fluorescence. **(E)** The revisable mitochondria contraction. **(F)** The irrevisable mitochondria contraction.

It is impossible to grasp the fast fission and fusion events without an active and visible readout phenomenon. Mitoflash, as a novel biomarker for mitochondrial respiration and activation, can be detected by circularly permuted YFP (cpYFP). It has been widely used to monitor mitochondria in different organisms (Gong et al., 2015a). Mitoflash can amplify the visualizable signal of activated mitochondria. Thus, highly active or dynamic mitochondria can be easily distinguished from other static mitochondria. We first successfully visualized real-time single mitochondrial fusion, fission, kissing, and contraction events in mature cardiomyocytes by monitoring thousands of cells (Qin et al., 2018). These mitochondrial dynamic events only occurred between adjacent mitochondria along the direction of the myofilament, other than the Z line, and occur less frequently (Figure 4). Eisner et al. (2017) demonstrated that mitochondrial fusion, as monitored by PAGFP, decreases markedly in cultured cells, as compared to the frequent mitochondrial fusion seen in freshly isolated rat ventricular myocytes, because of the decay in contractile activity. However, it is impossible to monitor single mitochondrial fusion and fission in freshly isolated beating myocytes.

In cultured adult cardiomyocytes, kissing and contraction, rather than common fission and fusion, are the main dynamic events. Among these events, the frequency of mitochondrial fusion is the lowest (Qin et al., 2018). The process from fission to fusion takes longer than the process from fusion to fission in other cell types (Twig et al., 2008).

Mitochondrial fusion and kissing allow mixing or exchange of mitochondrial matrix components among mitochondria. Thus, we propose that mature (adult) cardiomyocytes primarily depend on mitochondrial kissing and fusion to support the exchange of metabolic components between adjacent mitochondria. Qin et al. (2018), using mitoflash, found that most mitochondrial contractions were reversible and could recover in mature cardiomyocytes. This type of higher-frequency reversible contraction may promote the internal compartment metabolic component exchange of individual mitochondria (Qin et al., 2018).

## Mitochondrial Dynamics and Heart Disease

Cardiovascular diseases are characterized by high morbidity, disability, and mortality rates. They cannot be completely cured, according to clinical trials, or are prone to relapse after treatment. Abnormalities in cardiac energy metabolism are a critical determinant of heart failure (Ashrafian et al., 2007). In this regard, mitochondria are a crucial energy factory for the heart and produce 6 kg/day of ATP to maintain about 1,000 heartbeats per day (Neubauer, 2007). Mitochondrial function is central to the physiology and pathology of the adult heart. It is widely accepted that there is a complex interplay between mitochondrial dynamics and embryonic development, autophagy, and metabolism (Ong et al., 2012; Gottlieb and Bernstein, 2015). In the heart, chemical energy, such as fatty acids, is converted into the mechanical power of the actin–myosin interaction of myofibrils, which requires healthy mitochondria. If mitochondria fail to supply adequate energy for cardiac metabolism, heart failure may emerge. Mitochondrial dynamics is among the essential mechanisms of mitochondrial quality control. Conditional cardiac-specific Cre transgene-mediated Mfn1/Mfn2/Drp1 triple gene deletion leads to death in half of the mouse lines and survival of mice with decreased contractile function and senescent mitochondria (Song et al., 2017).

Next, we will focus on mitochondrial dynamics to introduce mitochondrial therapeutic targets in heart disease.

### Diabetic Cardiomyopathy

Patients with diabetes have high blood sugar levels; their blood vessels are more easily damaged and more likely to develop cardiovascular disease. Montaigne et al. (2014) investigated 141 patients and observed that the myocardial contraction of patients with diabetes was significantly worse than that of patients with obesity. They also found increased mitochondrial fragmentation and decreased expression of Mfn1, Mfn2, and OPA1. The disturbed mitochondrial function in patients with diabetes suggests that an imbalance of mitochondrial dynamics in cardiomyocytes might be a mechanism of cardiac dysfunction in diabetes. It has been observed that mitochondrial dynamics in individuals with type 2 diabetes (T2D) are disturbed (Zorzano et al., 2009). Diaz-Morales et al. (2016) compared patients with T2D and healthy subjects and found that the patients with T2D have reduced expression of Mfn1, Mfn2, and OPA1 proteins. In contrast to these fusion proteins, expression of Fis1, a fission protein, was significantly upregulated (Diaz-Morales et al., 2016). The same result was also found in obese Zucker rats, where both mRNA and protein levels of Mfn2 were reduced in skeletal muscles (Bach et al., 2005). It has been speculated that reduced Mfn2 expression may be related to impaired mitochondrial function in skeletal muscle (Zorzano et al., 2009). Moreover, mitochondrial morphology is different in T2D and lean subjects (Kelley et al., 2002). Compared with lean subjects, skeletal muscle mitochondria in patients with T2D become smaller and have large vacuoles under electron microscopy. Another study demonstrated that reduced Drp1 activation is beneficial to substrate metabolism and insulin resistance (Fealy et al., 2014). It has been verified that Drp1 expression is abnormal in the heart of db/db mice, and reducing excess fission through exercise can produce cardiovascular benefits (Veeranki et al., 2016). Vascular diseases, particularly coronary artery disease and stroke, are the major common causes of death in people with diabetes (Einarson et al., 2018). In 2004, Chen et al. (2004) first identified Mfn2 as a novel hyperplasia suppressor gene (HSG) reduced in hyperproliferative vascular smooth muscle cells (VSMCs) and capable of inhibiting VSMC proliferation (Twig et al., 2008; Dorn, 2013). Diabetes and cardiovascular disease are thus closely related to an imbalance between mitochondrial fission and fusion.

### Cardiac Hypertrophy and Heart Failure

Pathological cardiac hypertrophy is the growth response of the heart to an increase in mechanical stress induced by extrinsic factors, such as hypertension, and intrinsic factors, such as ischemia-induced cardiac remodeling or hypertrophic cardiomyopathy. Severe pathological hypertrophy and myocardial fibrosis eventually develop into heart failure (HF), a complex chronic clinical syndrome. Myocardial fibrosis leads to abnormal left ventricular function, which further results in HF (Moreo et al., 2009). Using cardiac Drp1 knockout mice and the cardiac Mfn double-knockout mice, it has been shown that inhibition of mitochondrial fission, but not fusion, occurs in adult hearts with increased myocardial fibrosis, provoking cardiomyocyte necrosis (Song et al., 2015). Energy deficit caused by mitochondrial dysfunction and abnormal metabolism is a critical factor in myocardial hypertrophy and HF (Rosca et al., 2013; Gottlieb and Bernstein, 2015). Most studies have indicated that myocardial infarction and HF are related to excessive mitochondrial fission and insufficient mitochondrial fusion (Chen et al., 2009; Ong et al., 2010). Compared with healthy hearts, mitochondria in rats with failing hearts are smaller and more fragmented (Hilfiker-Kleiner et al., 2006; Siasos et al., 2018). Changes in Mfn2 expression in cardiac hypertrophy have been studied in several disease models—from spontaneously hypertensive rats to pressure-overload hypertrophy caused by transverse aortic constriction (TAC) (Fang et al., 2007; Givvimani et al., 2014; Hall et al., 2016). On the one hand, Mfn2 gene expression was downregulated by ca. 80% in 10-month-old spontaneously hypertensive rats, as compared to the control group, while the ratios of heart weight to body weight and atrial natriuretic peptide (ANP) expression increased markedly (Chen et al., 2004; Fang et al., 2007). Similar results were found in mice with TAC, where Mfn2 expression was also reduced by 45% at 1 week and by 52% at 3 weeks (Fang et al., 2007). On the other hand, overexpression of cardiac Mfn2 could attenuate angiotensin II-induced myocardial hypertrophy (Yu et al., 2010). These results indicate the importance of fusion proteins in maintaining heart function. Mice with different conditional cardiac ablation of fusion proteins have been used to study the function of Mfn1 and Mfn2. Conditional cardiac ablation of both Mfn isoforms simultaneously has deleterious effects on mitochondrial morphology, respiration, and contractile function, resulting in death from cardiac failure (Eisner et al., 2017; Dorn, 2020). However, total cardiac knockout of Mfn2 and one Mfn1 allele (leaving one Mfn1 allele intact) was compatible with life, resulting in normal viability and baseline cardiac function. Conversely, total cardiac knockout of Mfn1, and one Mfn2 allele, leaving only one functional Mfn2 allele, evoked severe cardiomyopathy at baseline (Dorn et al., 2015). The different results of single allele knockout in Mfn proteins suggest that Mfn1 may be the key regulator of mitochondrial fusion activity, while Mfn2 may have other functions. Papanicolaou et al. (2011) provided evidence for this hypothesis. They found that mitochondria in Mfn-1-deficient hearts were smaller than those in Mfn-2-deficient hearts, and the proportion of enlarged mitochondria in Mfn-2-deficient hearts became more tolerant to Ca^2+^-induced mitochondrial permeability transition pore (MPTP) opening (Fang et al., 2007). Our investigations indicated that Mfn2 overexpression could maintain cardiac mitochondrial function by increasing mitochondrial biogenesis from mitochondrial dysfunction-induced cardiotoxicity (Qin et al., 2020). It has been reported that Mfn2 also plays a vital role in Parkin-mediated mitochondrial autophagy, which is essential for mitochondrial quality control and cardioprotection (Gong et al., 2015b; Song and Dorn, 2015). Some reports have revealed that the role of Parkin inadequately eliminating damaged mitochondria is essential for myocardial function after infarction (Huang et al., 2011; Papanicolaou et al., 2011). OPA1 also participates in maintaining cardiomyocyte homeostasis (Wu et al., 2019). The OPA1-deficient mouse more easily develops myocardial hypertrophy, independent of MPTP opening, indicating that the function of Mfn proteins in the adult mouse is different from that in the suckling mouse (Chen et al., 2012; Wai et al., 2015). Overexpression of OPA1 normalized mitochondrial quality control and sustained cardiomyocyte function under hypoxic conditions (Xin et al., 2020). Chen et al. (2004) extracted OPA1 from explanted failing human heart samples and from a rat HF model and found that OPA1 protein levels were significantly reduced, even though the gene and protein levels of Mfn1 and Mfn2 remained unchanged. Decreased OPA1 in both failing human and rat hearts suggests an essential role for OPA1 in the progressive deterioration of the failing heart, particularly in ischemia-induced HF. Body weight and heart contractile ability were not influenced in OPA1^+/−^ mice, but the mitochondrial structure was altered, and the arrangement became irregular (Piquereau et al., 2012). In OPA1^+/−^ cardiomyocytes, the number of mitochondria was decreased overall. Interestingly, the proportion of large mitochondria (>1.8 μm^3^) was increased and that of small mitochondria (<1 μm^3^) was decreased (Piquereau et al., 2012). These results indicate that partial deficiency in OPA1 affects individual mitochondrial morphology and increases mitochondrial volume. The increase in the volume of mitochondria under partial deficiency of OPA1 is contrary to previous studies that showed that decreased mitochondrial fusion proteins results in mitochondrial fragmentation. Furthermore, elongated mitochondria were also found in neonatal cardiomyocytes with decreased OPA1 protein (Makino et al., 2011). Currently, therapies for HF mainly focus on mitochondrial biogenesis and oxidative stress (Szeto et al., 2011; Ramirez-Sanchez et al., 2013; Zhang et al., 2014). There have been few studies on small-molecule compounds or drugs specifically targeting mitochondrial fusion proteins in HF. This may be attributed to the pleiotropic non-fusion functions of Mfn2 and OPA1, which may play cardioprotective roles.

### Ischemia–Reperfusion Injury

In 2006, Brady et al. (2006) first found that mitochondrial shape changed from elongated and branched before ischemia to fragmented during ischemia and reperfusion in HL-1 cells (2 h of simulated ischemia and 90 min of reperfusion). Mitochondrial fragmentation may be a characteristic event during ischemia–reperfusion injury (IRI). Moreover, inhibition of fusion or promotion of fission can result in excessively fragmented mitochondria (Cipolat et al., 2004; Brady et al., 2006; Chang and Blackstone, 2010). Drp1 is an essential protein that modulates abnormal mitochondrial fission to generate a healthy and an unhealthy daughter mitochondria; the latter is removed by mitophagy to avoid accumulation of unhealthy mitochondria (Elgass et al., 2013). By combining Drp1 and cardiac myocyte-targeted Cre alleles in mice with cardiac Drp1 knockout, it was shown that the inhibition of fission ultimately resulted in a loss of cardiac myocyte mitochondria and lethal dilated cardiomyopathy (Dorn et al., 2015; Qin et al., 2020). In IRI, Drp1 activity is influenced by calcineurin, a heterodimeric protein involved in the transduction of a variety of Ca^2+^-mediated signals (Zou et al., 2001; Cribbs and Strack, 2007). During acute myocardial ischemia and reperfusion, cellular metabolism switches between mitochondrial oxidative phosphorylation and anaerobic glycolysis, resulting in a change in pH and MPTP opening. The inhibition of MPTP opening during ischemia (decreased pH <7.0) and the opening of the MPTP during reperfusion (pH to <7.0) eventually resulted in further mitochondrial calcium overload (Hausenloy et al., 2003). On the other hand, MPTP opening can stimulate mitophagy in cultured cardiac myocytes, leading to decreased mitochondrial content and induction of cell necrosis (Carreira et al., 2010; Ostadal et al., 2019). Calcineurin activation facilitates Drp1 dephosphorylation at Ser^637^, leading to the increased recruitment of Drp1 to the OMM from the cytoplasm, which facilitates mitochondrial fission and cardiomyocyte apoptosis (Cereghetti et al., 2008; Chang and Blackstone, 2010). In support of this mechanism, use of a calcineurin inhibitor, FK506, reduced diastolic blood pressure and myocardial infarct size post-ischemia–reperfusion by inhibiting calcineurin-mediated dephosphorylation of Drp1 at Ser^637^ and mitochondrial fission (Sharp et al., 2014). Overexpression of Drp1 in animal cardiomyocytes induced mitochondrial fragmentation without affecting the expression of other mitochondrial dynamics proteins or the function of cardiomyocytes. The protein kinase Akt exerts a cardioprotective effect in IRI by increasing the number of elongated mitochondria (Ong et al., 2015). Inhibition of Drp1 to decrease mitochondrial fission is thus a beneficial therapy (Ong et al., 2010, 2015). Treatment with 50 μmol/L of the mitochondrial division inhibitor-1 (Mdivi-1), a Drp1 inhibitor, elongated mitochondria (>2 μm or 1 sarcomere in length) by 14.5% compared with myocardial ischemia (Ong et al., 2010). Sharp et al. (2014) also showed the protective effect of Mdivi-1, as evidenced by reduced mitochondrial ROS and cardiomyocyte apoptosis in isolated neonatal murine cardiomyocytes and adult rat hearts. However, there was no change in mitochondrial morphology, myocardial fibrosis, myocardial infarction size, and cell apoptosis by Mdivi-1 (1.2 mg/kg) treatment in a large animal model of acute myocardial infarction (Ong et al., 2019). The opposing results of Mdivi-1 may be related to the specificity of Drp1 targets, and the different doses used in the various animal models should also be considered. Recent studies have shown that when Drp1 is absent, or knocked out, mitochondrial respiration and ROS production remain inhibited (Bordt et al., 2017). The response of mitochondria to Mdivi-1 when Drp1 is lacking suggests that Mdivi-1 is not a specific Drp1 inhibitor, and it has off-target Drp1-independent mitochondrial effects (Bordt et al., 2017). After the discovery of Mdivi-1 by the Cassidy-Stone team, some other mitochondrial fission inhibitors have been identified. A small non-competitive dynamin GTPase inhibitor, Dynasore, is effective in preventing pathologic left ventricular end-diastolic pressure elevation and increases cardiomyocyte survival by inhibiting Drp1 in ischemia–reperfusion conditions (Macia et al., 2006; Gao et al., 2013). In 2020, Disatnik et al. (2013) designed a Drp1 inhibitor, P110, which specifically inhibits the interaction of Fis1 and Drp1. They found that intraperitoneal injection of P110 at the onset of reperfusion is effective in producing long-term benefits, as evidenced by improving mitochondrial oxygen consumption by 68% and reducing the cardiac fractional shortening (FS) after ischemic injury, but P110 had no effect on mitochondrial fission activity in normal cardiomyocytes (Disatnik et al., 2013). Two other compounds, Drpitor1 and Drpitor1a, also have therapeutic potential in IRI. Both of these are more specific than Mdivi-1 in inhibiting the GTPase activity of Drp1 without interfering with the GTPase of dynamin 1 (Wu et al., 2020). These results highlight the importance of the balance of mitochondrial dynamics.

## Conclusions

Single mitochondrial dynamic events occur at a low frequency in adult cardiomyocytes. Mitochondrial dynamics regulated by mitochondrial dynamics proteins play a very important role in the physiology and pathology of the heart. It is crucial to maintain the integrity of mitochondria and protect mtDNA in the heart. On the one hand, damaged mitochondria are segregated by fission and removed by mitophagy. On the other hand, when new mitochondria are generated, the mitochondrial pool remains in balance. If mitochondrial damage is too severe to maintain integrity, cell death and heart disease will ultimately occur. However, it should be kept in mind that the major purpose of mitochondrial dynamics is to maintain mitochondrial fitness to produce ATP as fuel for heart contraction. ATP production remains central to mitochondrial function. Dysfunctional mitochondria not only generate less ATP but also produce more ROS, which can result in irreversible damage to mtDNA and proteins. In the failing human heart, ATP production is markedly lower than in the normal heart.

## Author Contributions

AL and GG wrote the manuscript. MG and WJ prepared the images. YQ and GG revised the manuscript. All authors contributed to the article and approved the submitted version.

## Conflict of Interest

The authors declare that the research was conducted in the absence of any commercial or financial relationships that could be construed as a potential conflict of interest.
